# Transforming Gerontological Research by Meaningfully Engaging Persons Living With Dementia

**DOI:** 10.1093/geroni/igaa057.2584

**Published:** 2020-12-16

**Authors:** Michael Lepore, Richard Fortinsky

## Abstract

Whereas persons living with dementia have commonly been subjects of gerontological research, participation of persons with dementia in designing and conducting studies and in scientific research meetings has been rare in the United States. In recent years, person-centered research models have arisen which give persons with dementia and their caregivers core roles in the research enterprise. As “co-researchers” with academic/professional researchers, persons with dementia and their caregivers are engaged in all aspects of the research enterprise, jointly developing research questions and study designs, collecting and analyzing data, planning research meetings, and disseminating results. International studies have shown that conducting research in collaboration with the population that is being studied has potential to enhance the quality and appropriateness of research and has been identified as an essential component of studies examining the effectiveness of different approaches to care. This session spotlights innovative advances in gerontological research that meaningfully engages persons with dementia. First, the engagement of persons with dementia in scientific meetings is addressed drawing on the examples of the 2017 and 2020 National Research Summits on Care Services and Supports for Persons with Dementia and their Caregivers (i.e., Summits). Next, a study using a patient engagement framework for caregivers and individuals with mild cognitive impairment living at home is discussed. Finally, the Empowering Partnerships program, which prepares researchers, persons with dementia, and care partners to collaborate in conducting research is reviewed. Outcomes and challenges of these innovations are examined, and the need for academic/professional researcher roles to evolve is discussed.

